# The effects of asymmetric competition on the life history of Trinidadian guppies

**DOI:** 10.1111/ele.12563

**Published:** 2016-01-12

**Authors:** Ronald D. Bassar, Dylan Z. Childs, Mark Rees, Shripad Tuljapurkar, David N. Reznick, Tim Coulson

**Affiliations:** ^1^ Department of ZoologyUniversity of OxfordSouth Parks RoadOxfordOX1 3PSUK; ^2^ Department of Animal and Plant SciencesUniversity of SheffieldSheffieldUK; ^3^ Department of BiologyStanford UniversityPalo AltoCAUSA; ^4^ Department of BiologyUniversity of CaliforniaRiversideCAUSA

**Keywords:** Asymmetrical competition, contest competition, integral projection model, interaction surface, mesocosm, scramble competition, symmetrical competition

## Abstract

The effects of asymmetric interactions on population dynamics has been widely investigated, but there has been little work aimed at understanding how life history parameters like generation time, life expectancy and the variance in lifetime reproductive success are impacted by different types of competition. We develop a new framework for incorporating trait‐mediated density‐dependence into size‐structured models and use Trinidadian guppies to show how different types of competitive interactions impact life history parameters. Our results show the degree of symmetry in competitive interactions can have dramatic effects on the speed of the life history. For some vital rates, shifting the competitive superiority from small to large individuals resulted in a doubling of the generation time. Such large influences of competitive symmetry on the timescale of demographic processes, and hence evolution, highlights the interwoven nature of ecological and evolutionary processes and the importance of density‐dependence in understanding eco‐evolutionary dynamics.

## Introduction

Individuals rarely have equal competitive ability. For example larger individuals in many animal species are more likely to acquire territories, gain initial access to resources, or acquire a mate, compared to those that are smaller. Such extremely asymmetric interactions are at one end of a continuum (Nicholson 1954; Varley *et al*. 1973; Hassell 1975; Weiner 1990). Evenly balanced competition, or symmetric competition, is at the other end. Under pure symmetric competition, all individuals are equally competitive and so resources end up being shared equally (Hassell 1975). There has been considerable work using simple unstructured models to explore the population dynamical consequences of symmetric vs. asymmetric competition as well as competition that lies along the continuum between them (Maynard Smith & Slatkin 1973; Hassell 1975; Brännström & Sumpter 2005; Anazawa 2010, 2012). There has been much less work considering the life history consequences of different forms of competition and whether these depend upon which aspects of the demography are affected by competition.

The degree of symmetry in competitive interactions within a species can be captured with interaction surfaces. Interaction surfaces are continuous versions of interaction or competition coefficient matrices (Levins 1968) with the values of the surface describing interactions within a species. We use interaction surfaces that describe how the difference in body length between the interacting individuals influences the outcome of competition. Interaction surfaces describe the competitive effect of one individual on another. There has been considerable work in incorporating interaction matrices into community models (Levins 1968; Inouye 2001; Young 2004) and they appear in simple age or discrete‐stage structured models of single species (Caswell 2001). This body of work has revealed that the degree of competitive asymmetry can determine population dynamical patterns (de Roos & Persson 2003; Brännström & Sumpter 2005). What has not been investigated is whether asymmetry can alter the pace of the life history (e.g. generation time) and whether the dynamical consequences depend upon whether the competition effects occur through survival, fertility, development or investment in offspring.

Interaction surfaces capture two contrasting processes that influence the outcome of competition. First, they can capture the competitive effects of one type of individual on other types of individuals competing for the same resource(s). Interaction surfaces that capture this type of competition are monotonic in form, where the bigger the difference between two types of individuals, the larger the degree of asymmetry in competition. Second, they can capture resource partitioning (*sensu* Macarthur & Levins 1967; Werner & Gilliam 1984), where individuals exploit different resource types. In this study, we consider only interaction surfaces that assume individuals are competing for the same resources.

Our model consists of a single species, single‐sex (female only) integral projection model (IPM). IPMs are a class of structured model that can be used to simultaneously investigate the dynamics of populations, life histories and continuous characters (Easterling *et al*. 2000; Ellner & Rees 2006; Coulson *et al*. 2010). They consist of at least one continuous character, but can also include discrete characters such as age (Childs *et al*. 2003; Ellner & Rees 2006) or spatial location (Bassar *et al*. 2015b). Models can be stochastic (Childs *et al*. 2004; Ellner & Rees 2007; Rees & Ellner 2009) and density‐dependent (Adler *et al*. 2010; Bassar *et al*. 2013, 2015b) and be constructed for one or two sexes (Schindler *et al*. 2013; Traill *et al*. 2014). They can be used to address questions in life history theory, population and community ecology and eco‐evolutionary dynamics and can be analysed using a variety of tools commonly used in quantitative genetics (Coulson *et al*. 2010) or adaptive dynamics (Rees & Rose 2002; Childs *et al*. 2011; Bassar *et al*. 2013). To date, models have not incorporated interaction surfaces, with density‐dependent models working with either just population size or biomass (but see Adler *et al*. 2010). Here, we demonstrate how interaction surfaces can be incorporated into IPMs, develop a simple IPM for the Trinidadian guppy (*Poecilia reticulata*) and analyse how varying degrees of competitive asymmetries influences total population size, mean body length, mean and variance in generation time, mean and variance in life expectancy and variance in lifetime reproductive success.

## Methods

### Modelling framework

A simple single species, single‐sex (female only) IPM can be written:

(1) $$n\left(z^{\prime },t+1\right)=\int \left[G\left(z^{\prime }|z\right)S\left(z\right)+D\left(z^{\prime }|z\right)M\left(z\right)B\left(z\right)S\left(z\right)\right]n\left(z,t\right)dz.$$

The model is a discrete time population projection model with the population structured by a continuous trait, *z*. Here, we assume *z* is body length, but the model can be used to describe any continuous trait. The function, n(z,t), describes the density of individuals in the population at time *t*, such that $\int_{a}^{b}n\left(z,t\right)dz$ is the number of individuals between length *a* and *b*. *S*(*z*) and *B*(*z*), are continuous functions, respectively, describing the probability of an individual with body length *z* at the beginning of the interval surviving and reproducing at the end of the interval. *M*(*z*) is a continuous function describing the mean number of offspring produced by an individual with body length *z*. The model assumes that all reproducing individuals do so at the end of the interval, immediately preceding the next census. *G*(*z*′|*z*) is the conditional probability density function describing transitions from length *z* at time *t* to length *z*′ at time *t* + 1*. D*(*z*′|*z*) is the conditional probability density function describing the distribution of offspring body length *z*′ at time *t* + 1 produced by parents with body length *z* at time *t*. Each of these vital rates can be estimated using regression methods. For example the probability of survival or reproduction can be estimated from using a generalised linear model with a binomial error structure and a logit link function. In other words:

(2) logit(S(z))∼V(z).

Equation (2) is fitted to data and describes how the vital rate changes as a function of body length, *z*. The linear predictor, *V*, is often is just a simple linear function of body length:

(3) $$V\left(z\right)=\beta_{0}+\beta_{z}z.$$

Analogous calculations can be conducted for each vital rate.

Density‐dependence can be incorporated in to the model by making at least one function dependent on the number/density of individuals in the population. If the functional form of *V*(*z*) is a simple additive relationship, and if density‐dependence acts through total population size, then:

(4) $$V\left(z,N\right)=\beta_{0}+\beta_{z}z+\beta_{N}N,$$

where $N=\int n\left(z\right)dz$ is total population density. The parameter β_*N*_ describes how the vital rate is altered by a change in the density of the population. In this case, the action of density‐dependence is independent of the length of other individuals within the population. In other words, competition is assumed to be symmetric. Other functional forms of the vital rates are possible, and depend on the biology of the organism and the trait of the organism being measured (see Online Supplement). Our particular focus here is on the terms that describe the density‐dependent effects and on how these are determined by the distribution of body lengths in the population.

If the action of density‐dependence also depends on the distribution of lengths in the population then we need to weight individuals depending on their length, relative to the focal individual, *z*. The effect of competition is then determined by N∫α(z,x)p(x)dx, where $p\left(x\right)=n\left(x\right)N^{-1}$ is the probability density function for individuals with body length *x*. The function α(z,x) describes an interaction surface. Substituting this into eqn (4) gives:

(5) $$V\left(z,p,N\right)=\beta_{0}+\beta_{z}z+\beta_{N}N\int \alpha \left(z,x\right)p\left(x\right)dx.$$

The shape of the interaction surface depends upon how competition among individuals of different body lengths operates. When length determines the ability to acquire resources, the interaction surface increases or decreases with the body length of the competitor. A simple functional form for the interaction surface in this case is given by the following equation:

(6) $$\alpha \left(z,x\right)={\left(\frac{x}{z}\right)}^{\varphi }.$$

Similar functional forms have been used in other studies of the effects of size on competitive interactions (e.g. Geritz *et al*. 1999). Other forms are given in the Supplemental Online Materials. When φ = 0, α(z,x)=1 for all *z* and *x*, and resource use is independent of body length we have equation (4). When φ = 1 then the outcome of competition is proportional to the body length (termed relative size‐symmetric in the plant literature, Weiner 1990). However, φ in our model is a continuous variable and can take on any value allowing competitive interactions to lie on a gradient between symmetric and various degrees asymmetric competition. In all cases, the quantity $N_{z}=N\int {\left(\frac{x}{z}\right)}^{\varphi }p\left(x\right)dx$ gives the density of all individuals in the population in terms of equivalent density of individuals with body length *z*.

### Model analysis

For numerical solutions of the IPM we approximate the integro‐difference equation describing the per time step dynamics (eqn (1)) by a matrix (Easterling *et al*. 2000), using the midpoint rule for numerical integration. We used matrices of dimension 100 × 100. We then evaluated the influence of changes in competitive ability as a function of length on the vital rates themselves, total population size, mean and variance in body length, generation time, life expectancy at birth and the variance in lifetime reproductive success over a range values of φ (−2 to 2) (see Table 1 for formulas). The competition parameter, φ, was changed in one vital rate function at a time over the range −2 to 2, with φ in all other vital rates held constant at zero. In the Online Supplement, we present an analysis where φ was perturbed simultaneously in all the vital rates (Fig. S1). All quantities were calculated at equilibrium. The equilibrium state was found numerically by iterating the population through 400 time steps. In some cases, asymmetric competition can lead to cycling (e.g. Persson *et al*. 1998; de Roos & Persson 2003) and in these cases, we calculated only the vital rates, total population size, mean body length and the variance in body length at the stable equilibria of a composite map across two time steps (see Online Supplement). We did not calculate the other quantities because their calculation is not well defined in cyclical dynamics.

**Table 1:** Formulas for population descriptors

Descriptor	Formulas
Total population size	N = **e** ^*T*^ **n**
Mean trait value	$E\left[z\right]=z^{T}nN^{-1}$
Variance in trait value	$var\left[z\right]={\left[\left(z\circ I\right)z\right]}^{T}nN^{-1}-E{\left[z\right]}^{2}$
Equivalent density	$n_{z}=z^{-\varphi }{\left(x^{\varphi }\right)}^{T}n$
Generation time	
Mean age within trait^1^	$q_{\mu }=E\left[q_{i}\right]=\sum_{a}\left(ad^{T}FP^{a-1}R_{0}^{-1}\right)$
Of offspring with mean trait value	*T*_*c*,*μ*_ = **q**_*μ*_ **c**
Variance among stable cohort	$T_{c,trait.var}=\sum_{i}E{\left[q_{i}\right]}^{2}c_{i}-T_{c,\mu }^{2}$
Life expectancy	
Mean age within trait^2,3^	$l_{\mu }=\left(E\left[l_{i}\right]\ldots E\left[l_{s}\right]\right)=e^{T}U$
Of offspring with mean trait value	T_l,μ_ = **l**_*μ*_ **c**
Variance among stable cohort	$T_{l,trait.var}=\sum_{i}E{\left[l_{i}\right]}^{2}c_{i}-{T_{l,\mu }}^{2}$
Lifetime reproductive success	
Mean offspring within trait^2^	$r_{\mu }=\left(E\left[r_{i}\right]\ldots E\left[r_{s}\right]\right)=e^{T}\mathrm{FU}$
Of offspring with mean trait value	R_*μ*_ = **r**_*μ*_ **c**
Variance among stable cohort	$R_{trait.var}=\sum_{i}E{\left[r_{i}\right]}^{2}c_{i}-R_{\mu }^{2}$

**n** is a vector containing the number of individuals of each length. **e** is a vector of 1s. $U={\left(I-P\right)}^{-1}={\left(I-\mathrm{GS}\right)}^{-1}$ is the fundamental matrix. *R*_0_ is the dominant eigenvalue of the generation matrix ($A_{0}=\sum_{a}FP^{a-1}$, (for details, see Steiner *et al*. 2014)), where **F** = **DMBS**. **d** and **c** are the left and right eigenvectors (respectively) of **A**_0_. The vector **c** is scaled so that $1=\sum_{i}c_{i}$. **d** is normalised so that $\left(d,c\right)=1$. The superscript *T* denotes the transpose. $E\left[\;\right]$ is the expected value. For example $E\left[q_{i}\right]$ is the mean age of reproduction of offspring born with trait value *i* (generation time). Weighting the vector of means, **q**_*μ*_, by the stage structure of the stable cohort (**c**) then gives the mean age of reproduction of the mean offspring at birth, *T*_*c*,*μ*_. References: ^1^Steiner *et al*. (2014), ^2^Steiner & Tuljapurkar (2012), ^3^Caswell (2001).

### Study species

We demonstrate how different types of competition may influence ecological and demographical quantities by building and parameterising an IPM of Trinidadian guppies (demographical data from Bassar *et al*. (2013)). Most vital rates in guppies are functions of body length measured as standard length (SL), which is the linear distance between the tip of the snout to the posterior end of hypural plate in the tail. Results from empirical density manipulations in natural streams and factorial experiments in mesocosms have revealed that vital rates depend on density (Reznick *et al*. 2012; Bassar *et al*. 2013) and also on the mean body length (Bassar *et al*. 2015a). The data we use have previously been analysed and published to identify effects of density‐dependence and whether the density‐dependent response differs among populations that live with and without predators. Here, we re‐analyse data from fish captured from predator‐free streams only. We provide brief details of experimental design and data collection in the Online Supplement and refer readers who desire further detail to Bassar *et al*. (2013).

### Model parameterisation

The model for guppies is a single‐sex (female only) model. The time step for the guppy IPM is 28 days, which is slightly longer than one female reproductive cycle. In natural populations, individuals are at different stages of pregnancy and so offspring are continuously being produced across individuals in the population. Using a discrete time model to approximate this process has little effect on the outcome (Bassar *et al*. 2013). The length interval over which the projection is calculated is 2–35 mm SL. Although guppies are never smaller than 5 mm at birth, decreasing the lower limit prevents boundary effects. Likewise, wild guppies from these populations rarely obtain 30 mm in length and the extreme upper limit prevents the chances of boundary effects influencing results. All the vital rates are functions of standard length (SL) and density (Table S1).

Parameters for each of the vital rate functions were estimated using either linear mixed or generalised linear mixed models. When estimable, mesocosm number and river drainage of origin was included as a random effect. The models were fitted assuming competition is symmetric (φ = 0). Although this does not necessarily provide the best statistical fit for these data, our aim was to examine how changing φ, and hence the nature of the competitive interactions, alters population descriptors.

## Results

Increased density decreased somatic growth, survival, the probability of becoming pregnant, and if pregnant, the number of offspring being carried (Bassar *et al*. 2013 and Table S3). In contrast, increased density increased the length of offspring at birth (Bassar *et al*. 2013 and Table S3). These effects were estimated assuming length‐independent competition among individuals (φ = 0). We next describe the population dynamic consequences of changing the nature of competition from a scenario where smaller individuals are competitively superior (φ < 0) to one wherein larger individuals are competitively superior (φ > 0) in each function within our IPM.

**Figure 1:**
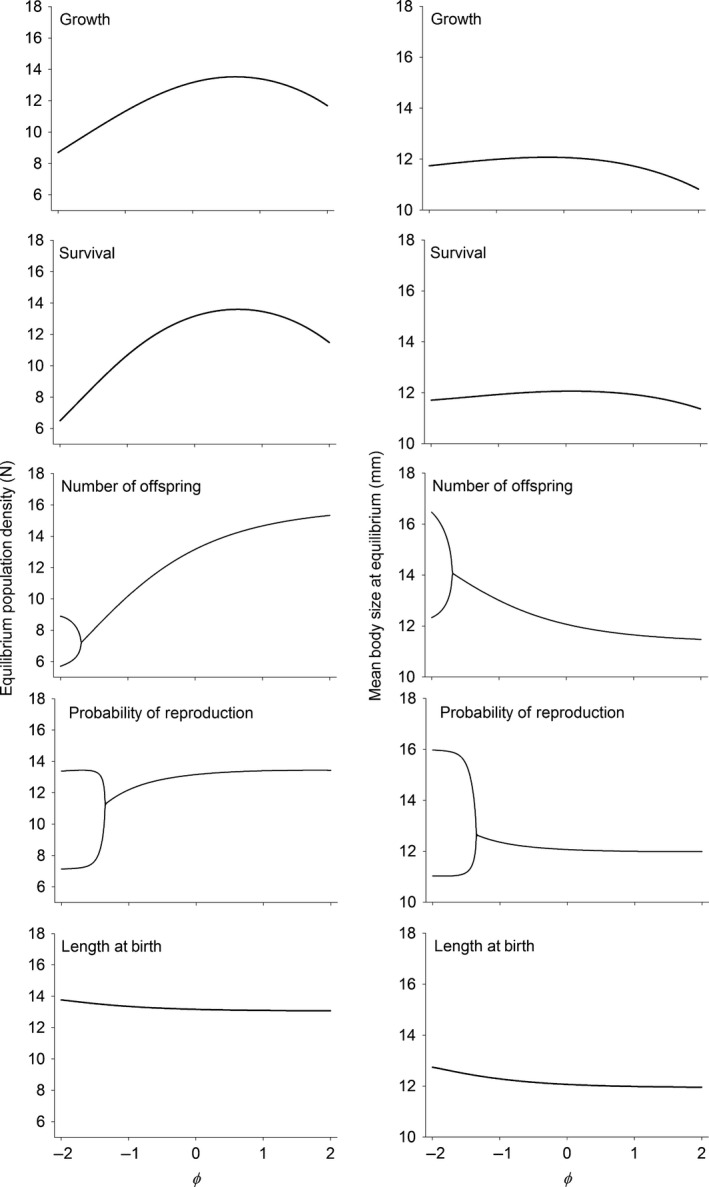
Equilibrium population density and mean body length with φ is assumed to varying between −2 and 2. Values were obtained using the formulas in Table 1 after numerically finding the equilibrium. For the number of offspring and the probability of reproduction, lower values of φ destabilised the equilibrium leading to a two‐period cycle.

At the population level, manipulating the symmetry of competition through somatic growth, survival and offspring length led to single, stable equilibria across the range of φ values (−2 to 2) (Fig. 1). In somatic growth and survival these changes led to non‐monotonic changes in population density with maximum values when larger individuals were slightly more competitive (φ value between 0.5 and 1). Changing competitive interactions along the same spectrum in offspring length led to a monotonically decreasing equilibrium population size (Fig. 1).

The consequences of changing the symmetry of competition operating via the number of offspring and the probability of reproduction were qualitatively very different compared to the other vital rates. When smaller individuals had an extreme competitive advantage over larger individuals (values of φ less than about −1.60) changes in the number of offspring led to a destabilisation of the equilibrium and a transition into a stable two‐period cycle (Fig. 1). Similarly, a competitive advantage of smaller over larger individuals (values of φ less than about −1.32) in the probability of reproduction also led to the destabilisation of the equilibrium and a transition into a stable two‐period cycle (Fig. 1). In both cases, the two‐period cycle meant that the population cycled between a high‐density population with smaller mean body length and a lower density population with larger mean body length. Increasing φ above the bifurcation monotonically increased equilibrium population density and monotonically decreased mean body length (Fig. 1). Similarly, increasing φ above the bifurcation in the probability of reproduction monotonically increased equilibrium population size and monotonically decreased mean body length (Fig. 1).

**Figure 2:**
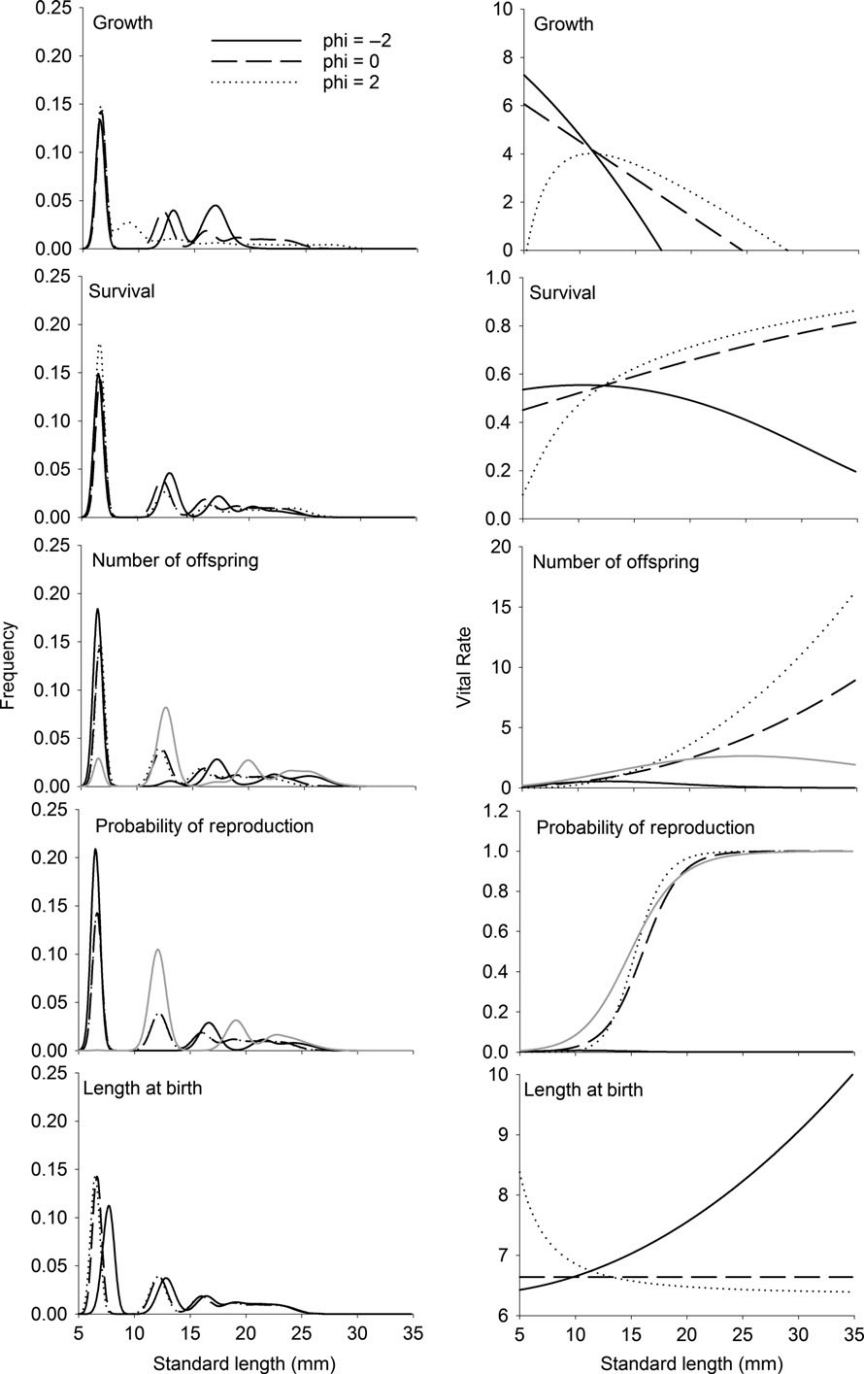
Stable length distributions and vital rates at equilibrium with φ assumed equal to −2, 0 and 2. Solid black and solid grey for number of offspring represent the stable length distribution and vital rates at each of the equilibria in the composite map.

The effects of competitive interactions on the equilibrium population density and length distribution arise in two ways. First, changing the nature of the competitive interactions alters how the vital rates respond to the distribution of lengths in the population. This change can in part be seen by examining the relationships between the values of the vital rates and body length for different values of φ. When smaller individuals had a competitive advantage (φ < 0) or if competitive ability was not a function of body length (φ = 0), somatic growth was a monotonically decreasing function of initial length (Fig. 2). This partially reflects faster growth in small guppies compared to larger ones, independent of food acquisition. However, if larger individuals are competitively superior, then the relationship between initial length and somatic growth had a very different shape – becoming a non‐monotonic function with a maximum somatic growth rate around 10 mm (Fig. 2). The shape of the relationships between initial length and survival were such that if larger individuals were superior competitors (φ > 0) or if there is no relationship between length and competitive ability (φ = 0), then larger individuals should have higher survival (Fig. 2). However, if smaller individuals had a competitive advantage (φ < 0), then at some point, the relationship between length and survival flips such that smaller individuals have higher survival (Fig. 2).

Under symmetrical competition (φ = 0), offspring length at birth was independent of parent length. However, if smaller individuals were assumed to have a competitive advantage, then mean length of offspring at birth increased with increasing parental length. The opposite pattern occurred if we assumed that larger individuals were competitively superior (Fig. 2). When competition was manipulated through the effects on the number of offspring there were very few offspring produced in one of the periods (grey line in Fig. 2) followed by increased numbers in the following period (black lines in Fig. 2). When competition was manipulated in the probability of reproduction, one of the periods was characterised by a near complete failure of individuals of all lengths to reproduce (grey lines in Fig. 2). In contrast in the following period, the probability of reproduction was higher (black lines in Fig. 2).

Changing the symmetry of the competitive interaction also alters the distribution of lengths in the population itself. Moving through the scenarios where smaller guppies are competitively superior (φ < 0), to where competition is independent of length (φ = 0), to the scenario where larger individuals are competitively superior (φ > 0) for somatic growth leads to a compression of the stable length distribution towards smaller lengths at equilibrium (Fig. 2). A similar pattern, but smaller in magnitude, was seen for survival. When these competitive asymmetries were manifested through offspring length at birth the stable length distribution was mostly unaffected moving from φ = 0 to when larger individuals were competitively superior (φ > 0). However, if the smaller individuals were competitively superior (φ < 0) then the mean size of the smallest individuals increased. With extreme competitive superiority of smaller fish in the number of offspring and the probability of reproduction lead to cohort cycling. That is the length distributions fluctuated between having very few or almost no small fish to many of the small fish (compare solid grey and black lines in Fig. 2).

**Figure 3:**
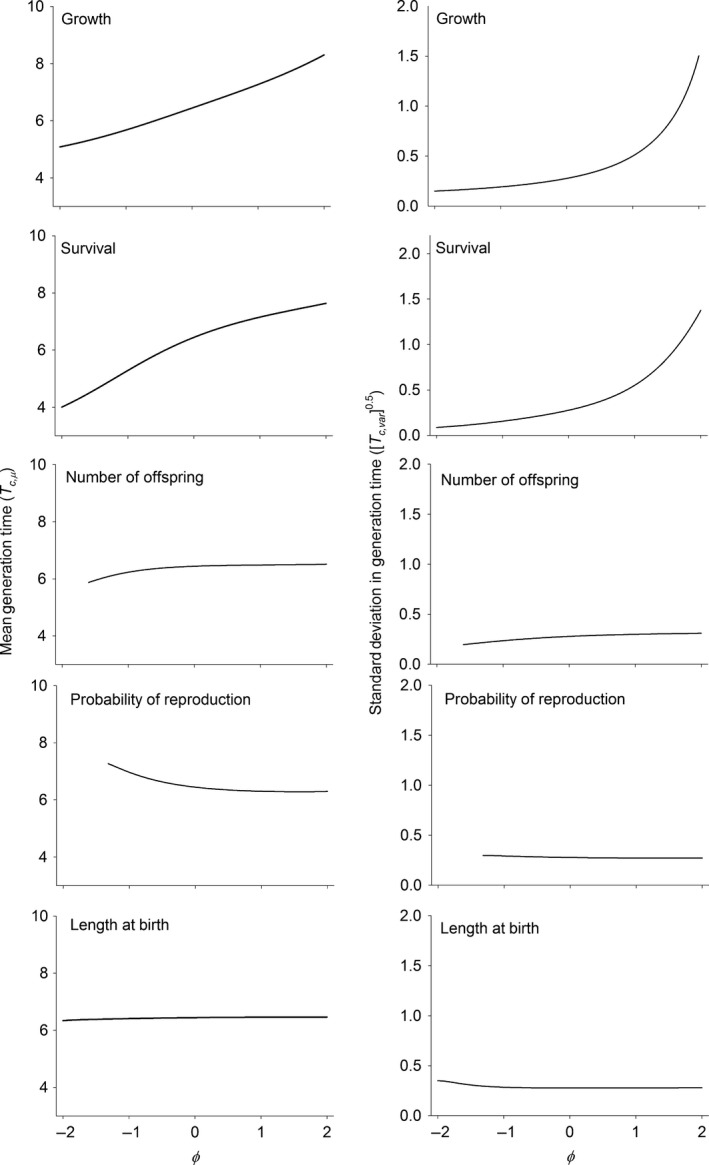
Mean and trait‐based standard deviation in the generation time (age of reproduction of a cohort) as a function of φ in each of the vital rates. φ is a measure of the degree of asymmetry in competitive interactions. Negative values of φ indicate that smaller sized individuals have a competitive advantage over larger sized individuals. Zero means that competitive ability does not depend on the trait value (symmetrical competition). φ values greater than zero indicate that larger individuals are competitively superior to smaller individuals. Measures below the bifurcation are omitted because the calculation of the generation time is not well‐defined for cyclical dynamics. See text and Table 1 for calculating trait‐based variances.

These changes in the symmetry of competition also had large effects on the speed of the life history and the distribution of lifetime reproductive success within the population. One way to measure the speed of the life history is using generation time (mean age of reproduction of a cohort). For all the vital rates except the probability of reproduction, moving from asymmetric competition with competitively superior smaller individuals (φ < 0) to symmetric competition (φ = 0) to asymmetric competition with competitively superior larger individuals (φ > 0) slowed the life history as measured by the generation time. In somatic growth and survival these changes were dramatic with a near doubling of the generation time from one extreme competitive asymmetry to the other extreme (Fig. 3). Comparatively, shifts in length‐based competitive ability acting through the probability of reproduction had a much smaller and opposite effect on the speed of the life history (Fig. 3).

The effect of changes in the nature of competition also influenced the variance in generation time (Fig. 3). Below and in the figures we use the standard deviation because it facilitates comparison with the mean which has the same units. For somatic growth and survival, the standard deviation increased in an approximately exponential fashion with increasing values of φ. Patterns in the number of offspring and the probability of reproduction mostly mirrored the effects on the mean. The influence of competitive interaction through offspring length was flat over much of the range of φ values, but slightly increased for the most negative values (Fig. 3).

**Figure 4:**
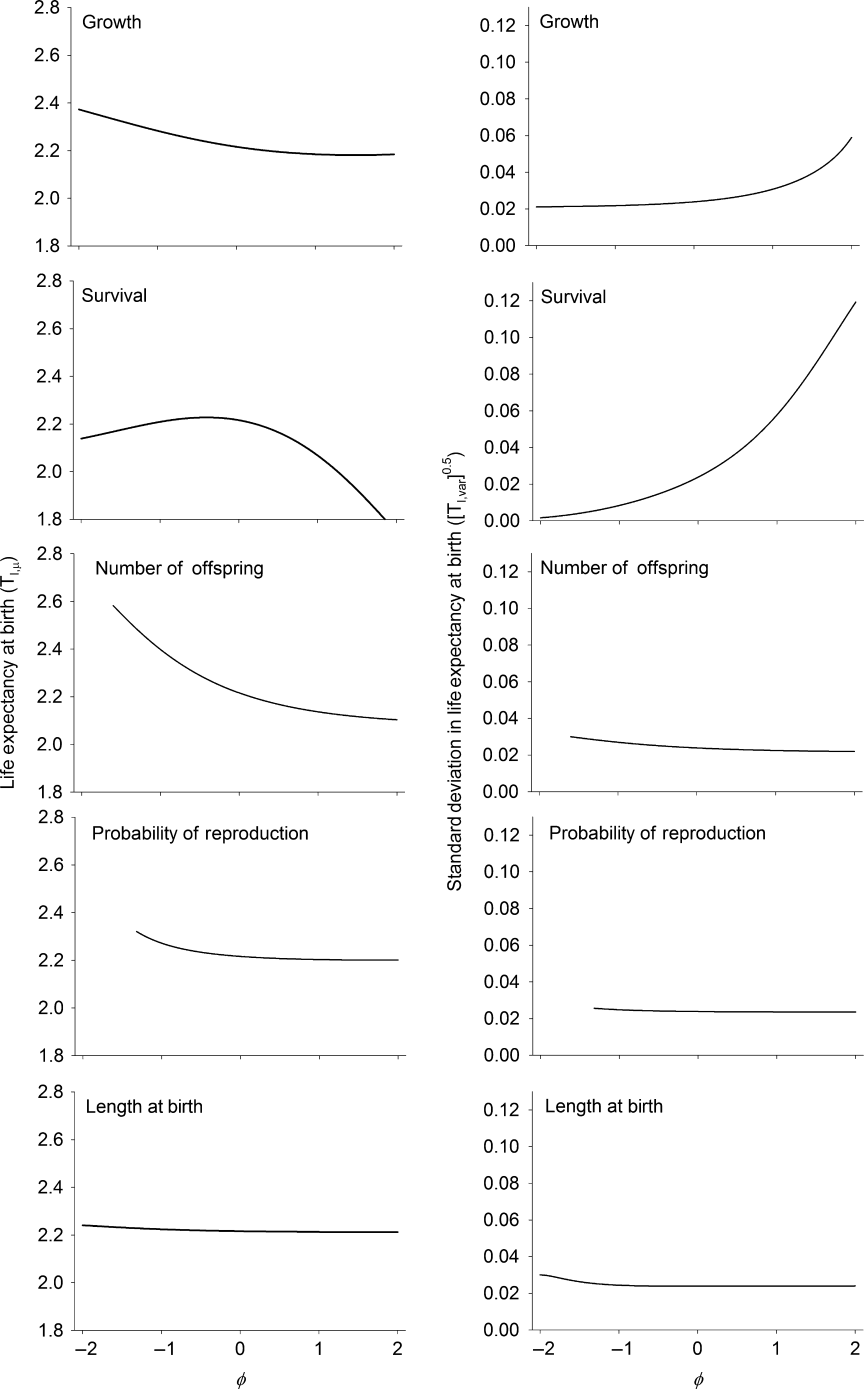
Mean and trait‐based standard deviation in life expectancy as a function of φ in each of the vital rates. φ is a measure of the degree of asymmetry in competitive interactions. Negative values of φ indicate that smaller sized individuals have a competitive advantage over larger sized individuals. Zero means that competitive ability does not depend on the trait value (symmetrical competition). φ values greater than zero indicate that larger individuals are competitively superior to smaller individuals. Measures below the bifurcation are omitted because the calculation of the life expectancy is not well‐defined for cyclical dynamics. See text and Table 1 for calculating the trait‐based variances.

Another measure of the speed of the life history is the life expectancy of the mean sized individual at birth. For all vital rates except survival, mean life expectancy of the average sized offspring at birth was longest when smaller guppies were competitively superior to larger guppies (φ < 0, Fig. 4). Increasing φ not only decreased mean life expectancy, but the slope of the relationship became shallower as competition moved from a symmetric (φ = 0) relationship between length and competitive outcome towards competitive interactions dominated by larger guppies (φ > 0). For survival, mean life expectancy initially increased, then decreased with changes in competitive interactions (Fig. 4).

The nature of the competitive interactions influenced the standard deviation in life expectancy among individuals born at different lengths. When competitive interactions were altered in somatic growth or survival, the standard deviation increased in a roughly exponential fashion with increasing values of φ (Fig. 4). Over much of the range of values of φ this meant that the mean and the standard deviation in life expectancy moved in opposite directions. In contrast, the effects were very small for the three vital rates related to reproduction (number of offspring, probability of reproduction and length at birth; Fig. 4).

**Figure 5:**
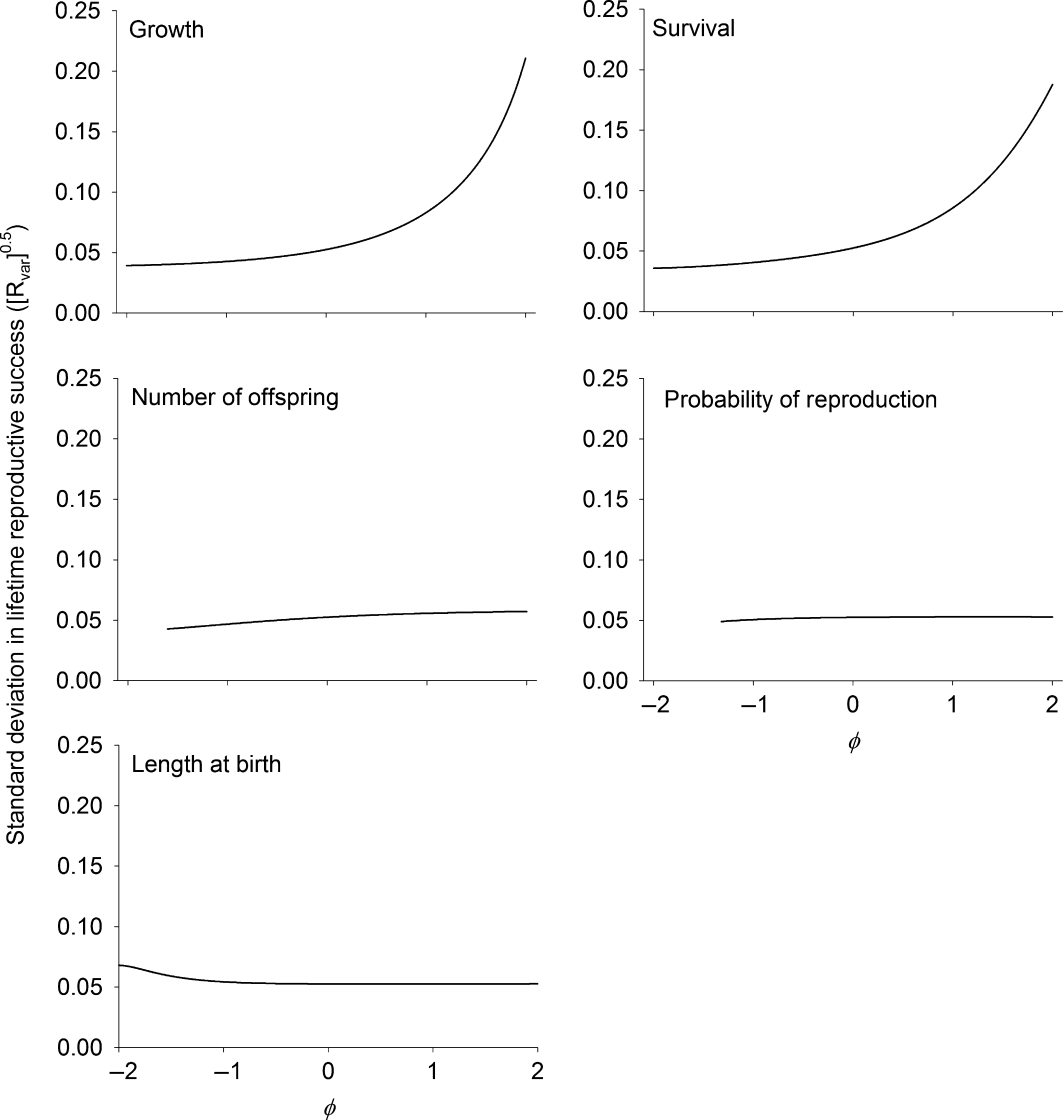
Trait‐based standard deviation in lifetime reproductive success as a function of φ in each of the vital rates. φ is a measure of the degree of asymmetry in competitive interactions. Negative values of φ indicate that smaller sized individuals have a competitive advantage over larger sized individuals. Zero means that competitive ability does not depend on the trait value (symmetrical competition). φ values greater than zero indicate that larger individuals are competitively superior to smaller individuals. Measures below the bifurcation are omitted because the calculation of generation time is not well‐defined for cyclical dynamics. See text and Table 1 for calculating the trait‐based variances.

All the competition scenarios presented here were evaluated at equilibrium (stable population density and size‐structure) and so mean lifetime reproductive success is unity. However, how lifetime reproductive success is distributed within the population was affected by the symmetry of competition. The standard deviation in lifetime reproductive success monotonically increased when competitive interactions moved from those where smaller individuals were competitively dominant (φ < 0) to when larger individuals were competitively dominant (φ > 0) in somatic growth, survival, the number of offspring, and the probability of reproduction (Fig. 5). In contrast, the standard deviation in lifetime reproductive success decreased when larger individuals were competitively dominant in the length of offspring at birth (Fig. 5). The largest changes in the standard deviation were again seen in survival and somatic growth where the standard deviation increased in an exponential‐like fashion with increasing values of φ.

## Discussion

Density‐dependence is an old concept in biology and different assumptions about the underlying mechanisms lead to very different population dynamics (Geritz & Kisdi 2004; Brännström & Sumpter 2005). For example different assumptions about the mechanism of competition lead to the logistic, Ricker and Beverton‐Holt models (Varley *et al*. 1973; Brännström & Sumpter 2005). However, many natural populations are structured by either continuous or discrete traits (Werner 1988) and in such cases simple models of density‐dependence will typically be insufficient to capture the dynamics. Our work develops a method to incorporate these different types of competitive interactions in discrete time continuous trait‐structured models that can easily be fit to data. We explore the extent to which different types of competitive interactions among individuals of different body lengths influences population densities, mean body lengths, the speed of the life history and the variance in reproductive success in the Trinidadian guppy.

A novel finding of the work we present here is that the symmetry of competition can alter both the mean and the variances among individuals in the speed of the life history measured as the generation time or life expectancy. Generation time and is an important quantity in population and evolutionary biology because it sets the timescale over which evolutionary change can occur (Charlesworth 1994). In all vital rates except the probability of reproduction, increasing asymmetry in competitive interactions such that larger individuals had a competitive advantage led to a slowing of the life history and hence an increase in the timescale over which evolutionary change is measured.

Whereas the mean generation time incorporates changes due to growth, survival and fertility, mean life expectancy tells us only about growth and survival. In contrast to mean generation time, life expectancy of an average sized individual at birth mostly decreased as the asymmetry in competitive interactions increasingly favoured larger individuals (φ > 0). Increasing mean generation time with decreasing life expectancy initially may occur if the amount of reproduction at later ages increases as early survival decreases. Previous authors have shown that the nature of density‐dependent interactions can alter the direction of evolutionary changes in the life history (e.g. Mylius & Diekmann 1995) and the results presented here suggest that the timescale over which evolution occurs is also strongly influenced by the degree of competitive asymmetry.

The variances in generation time and life expectancy tells us how much individuals are likely to vary the speed of the life history, given the variance in offspring length at birth. Both of these variances are generally small, which is a reflection of the small variance in body length at birth. Nevertheless, these variances strongly increased as the competitive advantage of larger individuals increased (φ > 0) in somatic growth and survival. Increasing the variance means that larger individuals at birth will have increasingly slower generation times and increasingly longer life expectancies.

Although competitive asymmetries manifested through the vital rates related to reproduction had little influence on the speed of the life history and the variance in reproductive success, they sometimes had large influences on the equilibrium population size and mean body length. Perhaps the most interesting of these effects was for the probability of reproduction and number of offspring. For these two vital rates, changes in the nature of competition led to a destabilisation of the equilibria and a shift towards to 2‐period cycle (Fig. 1). Persson *et al*. (1998) and de Roos & Persson (2003) have shown that competitive interactions among different sized individuals can lead to cycling. Their model is a continuous time physiologically structured model where higher attack rates by the juvenile class leads to population cycles. Such cohort cycling in their models is induced by a delay in the attainment of reproductive state. Under similar competitive scenarios, our results for the number of offspring and probability of survival also lead to dynamics that were characterised by near failure of reproduction in one of the two periods (Fig. 2). The consistency of the results for competitive dominance of smaller individuals between the approach of Persson *et al*. (1998) and de Roos & Persson (2003) and our different approach here means that it is unlikely that these dynamics are consequences of the types of models employed and rather are general biological predictions of what may occur under these competitive scenarios.

While the terms symmetrical and asymmetrical competition are widely used in the plant and animal literature, the terms scramble and contest competition are also often used in the animal literature (Varley *et al*. 1973; Lawton & Hassell 1981; Connell 1983; Weiner 1990). Under classical scramble competition all individuals share in the resources equally, and resultantly, the effect of increased density affects all individuals the same way – the interaction is symmetrical. Under classical contest competition individuals compete for a limiting resource and some individuals win and others lose the contest. The original formulation of contest competition made the unlikely assumption that individuals in the population are identical such that individuals win or lose contests independently of their traits. This convenience allowed earlier, unstructured models that did not explicitly model traits to include extreme competitive interactions where there are winners and losers. It is far more likely that the outcome of such competitive interactions depend on the trait values of the individuals engaged in the contest, and our modelling framework allows for these trait‐based competitive interactions to be incorporated explicitly into the models.

Importantly, in this study we do not estimate the competition parameter φ, rather we simply perturb its value to observe the effect it may have on the dynamics. In general, φ can be estimated when data are available where both the density and distribution of trait values in the population vary. This can been seen in the competition term in eqn (5) ($\beta_{N}N\int \alpha \left(z,x\right)p\left(x\right)dx$) where total population density and the distribution of lengths are multiplied by each other (i.e. they interact). How the interaction occurs then depends on the interaction surface. Whether the range of density and distribution variation is sufficient to estimate φ can and should be checked using simulated data (see Online Supplement for example) prior to fitting observational data or when designing and analysing an experiment. For guppies, doubling the density and manipulating mean body length is sufficient to estimate φ (see example in Online Supplement).

The model we developed here is for a single continuous trait – body length. However, the framework can be used for any continuous character that determines the outcome of competition and can easily be extended to include multiple traits. Our goal here was to demonstrate the importance of competitive asymmetries in structured populations and to provide a modelling framework that can be parameterised using data that is regularly collected by many ecologists. Incorporating competitive asymmetries into investigations hopefully will facilitate movement beyond simple characterisations of density‐dependence for a wide range of organisms and ultimately a broader understanding of the influence of competitive asymmetries on population dynamics and the pace of life.

## References

[ele12563-bib-0001] Adler, P.B. , Ellner, S.P. & Levine, J.M. (2010). Coexistence of perennial plants: an embarrassment of niches. Ecol. Lett., 13, 1019–1029. 20545729 10.1111/j.1461-0248.2010.01496.x

[ele12563-bib-0002] Anazawa, M. (2010). The mechanistic basis of discrete‐time population models: the role of resource partitioning and spatial aggregation. Theor. Popul. Biol., 77, 213–218. 20184909 10.1016/j.tpb.2010.02.005

[ele12563-bib-0003] Anazawa, M. (2012). Interspecific competition models derived from competition among individuals. Bull. Math. Biol., 74, 1580–1605. 22538977 10.1007/s11538-012-9726-0

[ele12563-bib-0004] Bassar, R.D. , López‐Sepulcre, A. , Reznick, D.N. & Travis, J. (2013). Experimental evidence for density‐dependent regulation and selection on Trinidadian guppy life histories. Am. Nat., 181, 25–38. 23234843 10.1086/668590

[ele12563-bib-0005] Bassar, R.D. , Heatherly, T.I. , Marshall, M.C. , Thomas, S.A. , Flecker, A.S. & Reznick, D.N. (2015a). Population size structure dependent fitness and ecosystem consequences in Trinidadian guppies. J. Anim. Ecol., 84, 955–968. 25704755 10.1111/1365-2656.12353

[ele12563-bib-0006] Bassar, R.D. , Letcher, B.H. , Nislow, K.H. & Whiteley, A.R. (2015b). Changes in seasonal climate outpace compensatory density‐dependence in eastern brook trout. Glob. Change Biol., doi: 10.1111/gcb.13135. 10.1111/gcb.13135 26490737

[ele12563-bib-0007] Brännström, Å. & Sumpter, D. (2005). The role of competition and clustering in population dynamics. Proc. R. Soc. Lond. B: Biol. Sci., 272, 2065–2072. 10.1098/rspb.2005.3185 PMC1559893 16191618

[ele12563-bib-0008] Caswell, H. (2001). Matrix Population Models, 2nd edn Sinauer Ass, Sunderland, MA.

[ele12563-bib-0009] Charlesworth, B. (1994). Evolution in Age‐Structured Populations. Cambridge University Press, Cambridge.

[ele12563-bib-0010] Childs, D.Z. , Rees, M. , Rose, K.E. , Grubb, P.J. & Ellner, S.P. (2003). Evolution of complex flowering strategies: an age‐ and size‐structured integral projection model. P. Roy. Soc. Lond. B. Bio., 270, 1829–1838. 10.1098/rspb.2003.2399 PMC1691450 12964986

[ele12563-bib-0011] Childs, D.Z. , Rees, M. , Rose, K.E. , Grubb, P.J. & Ellner, S.P. (2004). Evolution of size‐dependent flowering in a variable environment: construction and analysis of a stochastic integral projection model. P. Roy. Soc. Lond. B. Bio., 271, 425–434. 10.1098/rspb.2003.2597 PMC1691612 15101702

[ele12563-bib-0012] Childs, D.Z. , Coulson, T. , Pemberton, J.M. , Clutton‐Brock, T. & Rees, M. (2011). Predicting trait values and measuring selection in complex life histories: reproductive allocation decisions in Soay sheep. Ecol. Lett., 14, 985–992. 21790931 10.1111/j.1461-0248.2011.01657.x

[ele12563-bib-0013] Connell, J.H. (1983). On the prevalence and relative importance of interspecific competition: evidence from field experiments. Am. Nat., 122, 661–696.

[ele12563-bib-0014] Coulson, T. , Tuljapurkar, S. & Childs, D.Z. (2010). Using evolutionary demography to link life history theory, quantitative genetics and population ecology. J. Anim. Ecol., 79, 1226–1240. 20704627 10.1111/j.1365-2656.2010.01734.x PMC3017750

[ele12563-bib-0015] Easterling, M.R. , Ellner, S.P. & Dixon, P.M. (2000). Size‐specific sensitivity: applying a new structured population model. Ecology, 81, 694–708.

[ele12563-bib-0016] Ellner, S.P. & Rees, M. (2006). Integral projection models for species with complex demography. Am. Nat., 167, 410–428. 16673349 10.1086/499438

[ele12563-bib-0017] Ellner, S.P. & Rees, M. (2007). Stochastic stable population growth in integral projection models: theory and application. J. Math. Biol., 54, 227–256. 17123085 10.1007/s00285-006-0044-8

[ele12563-bib-0018] Geritz, S.A.H. & Kisdi, É. (2004). On the mechanistic underpinning of discrete‐time population models with complex dynamics. J. Theor. Biol., 228, 261–269. 15094020 10.1016/j.jtbi.2004.01.003

[ele12563-bib-0019] Geritz, S.A.H. , van der Meijden, E. & Metz, J.A.J. (1999). Evolutionary dynamics of seed size and competitive ability. Theor. Popul. Biol., 55, 324–343. 10366556 10.1006/tpbi.1998.1409

[ele12563-bib-0020] Hassell, M.P. (1975). Density‐dependence in single‐species populations. J. Anim. Ecol., 44, 283–295.

[ele12563-bib-0021] Inouye, B. (2001). Response surface experimental designs for investigating interspecific competition. Ecology, 82, 2696–2706.

[ele12563-bib-0022] Lawton, J.H. & Hassell, M.P. (1981). Asymmetrical competition in insects. Nature, 289, 793–795.

[ele12563-bib-0023] Levins, R. (1968). Evolution in Changing Environments. Princeton University Press, Princeton, NJ.

[ele12563-bib-0024] Macarthur, R. & Levins, R. (1967). The limiting similarity, convergence, and divergence of coexisting species. Am. Nat., 101, 377–385.

[ele12563-bib-0025] Maynard Smith, J. & Slatkin, M. (1973). The stability of predator‐prey systems. Ecology, 54, 384–391.

[ele12563-bib-0026] Mylius, S.D. & Diekmann, O. (1995). On evolutionarily stable life histories, optimization and the need to be specific about density dependence. Oikos, 74, 1–12.

[ele12563-bib-0027] Nicholson, A. (1954). An outline of the dynamics of animal populations. Aust. J. Zool., 2, 9–65.

[ele12563-bib-0028] Persson, L. , Leonardsson, K. , de Roos, A.M. , Gyllenberg, M. & Christensen, B. (1998). Ontogenetic scaling of foraging rates and the dynamics of a size‐structured consumer‐resource model. Theor. Popul. Biol., 54, 270–293. 9878605 10.1006/tpbi.1998.1380

[ele12563-bib-0029] Rees, M. & Ellner, S.P. (2009). Integral projection models for populations in temporally varying environments. Ecol. Monogr., 79, 575–594.

[ele12563-bib-0030] Rees, M. & Rose, K.E. (2002). Evolution of flowering strategies in Oenothera glazioviana: an integral projection model approach. Proc. R. Soc. Lond. B: Biol. Sci., 269, 1509–1515. 10.1098/rspb.2002.2037 PMC1691055 12137582

[ele12563-bib-0031] Reznick, D.N. , Bassar, R.D. , Travis, J. & Rodd, F.H. (2012). Life history evolution in guppies VIII: the demographics of density regulation in guppies (Poecilia reticulata). Evolution, 66, 2903–2915. 22946811 10.1111/j.1558-5646.2012.01650.x

[ele12563-bib-0032] de Roos, A.M. & Persson, L. (2003). Competition in size‐structured populations: mechanisms inducing cohort formation and population cycles. Theor. Popul. Biol., 63, 1–16. 12464491 10.1016/s0040-5809(02)00009-6

[ele12563-bib-0033] Schindler, S. , Neuhaus, P. , Gaillard, J.‐M. & Coulson, T. (2013). The influence of nonrandom mating on population growth. Am. Nat., 182, 28–41. 23778224 10.1086/670753

[ele12563-bib-0034] Steiner, U.K. & Tuljapurkar, S. (2012). Neutral theory for life histories and individual variability in fitness components. Proc. Natl Acad. Sci., 109, 4684–4689. 22392997 10.1073/pnas.1018096109 PMC3311353

[ele12563-bib-0035] Steiner, U.K. , Tuljapurkar, S. & Coulson, T. (2014). Generation time, net reproductive rate, and growth in stage‐age‐structured populations. Am. Nat., 183, 771–783. 24823821 10.1086/675894 PMC6601636

[ele12563-bib-0036] Traill, L.W. , Schindler, S. & Coulson, T. (2014). Demography, not inheritance, drives phenotypic change in hunted bighorn sheep. Proc. Natl Acad. Sci., 111, 13223–13228. 25114219 10.1073/pnas.1407508111 PMC4246946

[ele12563-bib-0037] Varley, G.C. , Gradwell, G.R. & Hassell, M.P. (1973). Insect Population Ecology. Blackwell Scientific, Oxford.

[ele12563-bib-0038] Weiner, J. (1990). Asymmetric competition in plant populations. Trend Ecol. Evol., 5, 360–364. 10.1016/0169-5347(90)90095-U 21232393

[ele12563-bib-0039] Werner, E.E. (1988). Size, scaling, and the evolution of complex life cycles In: Size‐Structured Populations: Ecology and Evolution (eds Ebenmen B. & Persson L.). Springer, Berlin, pp. 60–81.

[ele12563-bib-0040] Werner, E. & Gilliam, J.F. (1984). The ontogenetic niche and the species interactions in size‐structured populations. Annu. Rev. Ecol. Syst., 15, 393–425.

[ele12563-bib-0041] Young, K.A. (2004). Asymmetric competition, habitat selection, and niche overlap in juvenile salmonids. Ecology, 85, 134–149.

