# Supplementary material for: The effects of asymmetric competition on the life history of Trinidadian guppies
Source: Ecol Lett. 2016 Jan 12;19(3):268–78. doi: 10.1111/ele.12563 (PMC4991285; doi:10.1111/ele.12563)
Supplement: Supplementary file 1 [file ELE-19-268-s001.docx]

**Supplementary Materials**

**THE EFFECTS OF Asymmetric COMPETITION ON THE LIFE HISTORY OF TRINIDADIAN GUPPIES**

Ronald D. Bassar^1,†^, Dylan Z. Childs^2^, Mark Rees^2^, Shripad Tuljapurkar^3^, David Reznick and Tim Coulson^1^

^1^ Department of Zoology, South Parks Road, University of Oxford, OX1 3PS

^2^ Department of Animal and Plant Sciences, University of Sheffield

^3^ Department of Biology, Stanford University, Palo Alto, California

^4^ Department of Biology, University of California, Riverside, California

^†^ corresponding author – email: ronald.bassar@zoo.ox.ac.uk

**METHODS**

MESOCOSM DATA

The mesocosms are eight cinder-block structures (~3 m x 1 m) that are laterally subdivided to yield 16 independent mesocosms. The mesocosms were built alongside a natural stream to facilitate natural colonization of stream invertebrates. Water for the mesocosms comes from a natural spring on the hill above the mesocosm facility. The water is gravity fed through a series of three settling tanks to help remove large organic material that washes in during high flow events. The final settling tank is fitted with 16 ¾ inch garden hoses that supply water to each mesocosm from the same level in the tank. Ball valves at the end of the hoses allow the adjustment of the flow rates into each mesocosm. Prior to introducing guppies, the mesocosms are seeded with a sample of benthic organic material and stream invertebrates from the adjacent natural stream.

Guppies used in the experiments were captured from predator-free streams on the Guanapo and Aripo Rivers, brought back to the lab, measured for standard length (SL) to the nearest thousandth of a millimeter and marked using two coloured, subcutaneous elastomer implants. Guppies were placed in the mesocosms the following day at either low density (12 fish) or at high density (24 fish). Size structures were nearly identical among density treatments and were within the range observed among populations that live with predators and predator free environments. Experiments were run separately for the Guanapo and the Aripo Rivers. After 28 days, all fish were recaptured and again measured for standard length. They were then sacrificed using an overdose of MS-222 and preserved in 10 percent formalin. They were then processed in the laboratory to obtain measurements of the pregnancy status and, if pregnant, the number and length of developing offspring.

FIELD DATA

The influence of guppy density on guppy survival was obtained by manipulating the density of guppies in natural pool habitats in five predator-free streams. In each stream, three pools were chosen that were roughly equal in size, shape and canopy cover. All guppies were removed from each pool and brought to the field station where they were measured for standard length and marked using three coloured subcutaneous elastomer implants. The fish were returned to the pools according at either half, control or at a fifty percent increased densities. All fish were returned to the pools they were captured in with the exception of the fish that constituted the portion of the increased density treatment. They were also returned at the same length distribution that was observed at initial capture. After 25 days, the fish in the pools and all adjacent pools were captured.

cOMPOSITE MAP OF CYCLICAL DYNAMICS

Briefly, the descriptors of the population cycle will appear as equilibria in the composite map across two time steps (Otto & Day 2007 pages: 425-428):

|  | $\mathbf{n(}t+2\mathbf{)}=\left. \mathbf{A} \right\vert_{\mathbf{n(}t+1\mathbf{)}}\left. \mathbf{A} \right\vert_{\mathbf{n(}t\mathbf{)}}\mathbf{n(}t\mathbf{)}.$ | **7** |
| --- | --- | --- |

Quantities that describe these equilibria can then be calculated based on this composite projection by beginning the composite projection at each of the equilibria.

|  | ${\hat{\mathbf{n}}}_{\tau}=\left. \mathbf{A} \right\vert_{{\hat{\mathbf{n}}}_{\tau+1}}\left. \mathbf{A} \right\vert_{{\hat{\mathbf{n}}}_{\tau}}{\hat{\mathbf{n}}}_{\tau},$  ${\hat{\mathbf{n}}}_{\tau+1}=\left. \mathbf{A} \right\vert_{{\hat{\mathbf{n}}}_{\tau}}\left. \mathbf{A} \right\vert_{{\hat{\mathbf{n}}}_{\tau+1}}{\hat{\mathbf{n}}}_{\tau+1}.$ | **8** |
| --- | --- | --- |

Because the equilibria of the original model are also equilibria of the composite model (but are unstable) (Otto & Day 2007 pages: 425-428), we refer here to the equilibria associated with the period-2 cycle of the composite model as ${\hat{\mathbf{n}}}_{\tau}$ and ${\hat{\mathbf{n}}}_{\tau+1}$. Descriptions of the equilibria such as $N, E\left[ z \right],var\left[ z \right]$, the stable stage and reproductive value can then be calculated directly from $\left. \mathbf{A} \right|_{{\hat{\mathbf{n}}}_{\tau+1}}\left. \mathbf{A} \right|_{{\hat{\mathbf{n}}}_{\tau}}$ or $\left. \mathbf{A} \right|_{{\hat{\mathbf{n}}}_{\tau}}\left. \mathbf{A} \right|_{{\hat{\mathbf{n}}}_{\tau+1}}$ using the standard tools of matrix population projection model analysis (Caswell 2001).

Literature Cited

1.

Otto, S.P. & Day, T. (2007). *A biologist's guide to mathematical modeling in ecology*

*and evolution*. Princeton Univ. Press, Princeton.

2.

Caswell, H. (2001). *Matrix Population Models*. 2nd edn. Sinauer Ass., Sunderland, Ma.

| Table S1. Parameters definitions, vital rate functions and matrix equivalents. | | | | |
| --- | --- | --- | --- | --- |
| Quantity | | | Equation/parameter definition | Matrix |
| Trait-values of focal types | | | $z$ | $\mathbf{z}$ |
| Trait-values of competitors | | | $x$ | $\boldsymbol{x}$ |
| Vital rate intercept | | | $\beta_{0}$ | - |
| Density-independent effect of trait on vital rate | | | $\beta_{z}$ | - |
| Effect of density on vital rate | | | $\beta_{N}$ | - |
| Population size function | | | $n\left( x \right)$ | $\mathbf{n}$ |
| Competition parameter | | | $\varphi$ |  |
| Equivalent density | | | $N_{z}=N\int\left( \frac{x}{z} \right)^{\varphi}p\left( x \right)dx$ | $\mathbf{n}_{z}=\mathbf{z}^{\boldsymbol{-}\varphi}\left( \boldsymbol{x}^{\varphi} \right)^{\boldsymbol{T}}\mathbf{n}$ |
| Mean somatic growth increment | | | $\mu_{G}\left( z,p,N \right)=\beta_{0}+\beta_{z}z+\beta_{N}N_{z}$ |  |
| Variance in somatic growth | | | $\sigma_{G}^{2}$ |  |
| Probability of growing to length $z^{'}$ given length $z$ at start of interval | $G\left( z^{'}\vert z,p,N \right)=\frac{1}{\sqrt{2\Pi\sigma_{G}^{2}}}{e^{\frac{-\left( z^{'}-\left( \mu_{G}\left( z,p,N \right)+z \right) \right)}{2\sigma_{G}^{2}}}}^{2}$ | | | $\mathbf{G}$ |
| Survival | | $S\left( z,p,N \right)=invlogit\left( \beta_{0}+\beta_{z}z+\beta_{N}N_{z} \right)$ | | $\mathbf{S}$ |
| Probability of reproduction | | $B\left( z,p,N \right)=invlogit\left( \beta_{0}+\beta_{z}z+\beta_{N}N_{z} \right)$ | | $\mathbf{B}$ |
| Number of offspring | | $M\left( z,p,N \right)=\beta_{0}z^{\beta_{z}}e^{\beta_{N}N_{z}}$ | | $\mathbf{M}$ |
| Mean offspring length of female of length $z$ at start of interval | | $\mu_{D}\left( z,p,N \right)=\beta_{0}+\beta_{z}z+\beta_{N}N_{z}$ | |  |
| Variance in offspring length | | $\sigma_{D}^{2}$ | |  |
| Probability of female length $z$ at start of interval having offspring of length $z^{'}$ | | $D\left( z^{'}\vert z,p,N \right)=\frac{1}{\sqrt{2\Pi\sigma_{D}^{2}}}{e^{\frac{-\left( z^{'}-\mu_{D}\left( z,p,N \right) \right)}{2\sigma_{D}^{2}}}}^{2}$ | | $\mathbf{D}$ |
| Stage transition | | $P\left( z^{'}\vert z,p,N \right)=G\left( z^{'}\vert z,p,N \right) S\left( z,p,N \right)$ | | $\mathbf{P}$ |
| Fertility | | $F\left( z^{'}\vert z,p,N \right)=D(z^{'}\vert z,p,N)M(z,p,N)B(z,p,N)S(z,p,N)$ | | $\mathbf{F}$ |

$\sigma_{G}^{2}$and $\sigma_{G}^{2}$ are the residual variances in the growth and offspring length analyses, respectively.

| Table S2. Alternative general equations and density dependent term forms. | | | |
| --- | --- | --- | --- |
| Quantity | With Trait Value | With Density | Comments |
| General Equation |  |  |  |
| $V\left( z,p,N \right)=\beta_{0}+\beta_{z}z+\beta_{N}N\int\left( \frac{x}{z} \right)^{\varphi}p\left( x \right)dx$ | Linear | Linear |  |
| $V\left( z,p,N \right)=\left( \beta_{0}+\beta_{z}z \right)e^{\beta_{N}N\int\left( \frac{x}{z} \right)^{\varphi}p\left( x \right)dx}$ | Linear | Exponential |  |
| $V\left( z,p,N \right)=\beta_{0}z^{\beta_{z}}e^{\beta_{N}N\int\left( \frac{x}{z} \right)^{\varphi}p\left( x \right)dx}$ | Power | Exponential |  |
| $V\left( z,p,N \right)=\beta_{0}z^{\beta_{z}}\frac{1}{\beta_{N}N\int\left( \frac{x}{z} \right)^{\varphi}p\left( x \right)dx}$ | Power | Power | $\beta_{N}$ is subsumed in $\beta_{0}$ for estimation. |
| $V\left( z,p,N \right)=\left( \beta_{0}+\beta_{z}z \right)\frac{1}{\beta_{N}N\int\left( \frac{x}{z} \right)^{\varphi}p\left( x \right)dx}$ | Linear | Power | $\beta_{N}$ is subsumed in $\beta_{0}$ and $\beta_{z}$ for estimation. |
| $V\left( z,p,N \right)=\beta_{0}e^{\beta_{z}z}e^{\beta_{N}N\int\left( \frac{x}{z} \right)^{\varphi}p\left( x \right)dx}$ | Exponential | Exponential |  |
| Density term |  |  |  |
| $N\int\left( \frac{x}{z} \right)^{\varphi}p\left( x \right)dx$ |  |  | Proportional, scaled to the focal individual. |
| $N\int e^{\varphi\left( x-z \right)}p\left( x \right)dx$ |  |  | Exponential, scaled to the focal individual. |
| $N\int\frac{z^{\varphi}}{\bar{x^{\varphi}}}\frac{x^{\varphi}}{\bar{x^{\varphi}}}p\left( x \right)dx=\frac{z^{\varphi}}{\bar{x^{\varphi}}}N\int\frac{x^{\varphi}}{\bar{x^{\varphi}}}p\left( x \right)dx=\frac{z^{\varphi}}{\bar{x^{\varphi}}}N$ |  |  | Proportional, scaled to the mean trait value. |
|  |  |  |  |

| Table S3. Parameters and standard errors from mesocosm and field studies of Trinidadian guppies. | | | | | | | | | | | | | | |
| --- | --- | --- | --- | --- | --- | --- | --- | --- | --- | --- | --- | --- | --- | --- |
|  | Mean Growth | |  | Survival | |  | Fecundity | |  | Prob. of Repro | |  | Offspring Length | |
| Parameter | Est | SE |  | Est | SE |  | Est | SE |  | Est | SE |  | Est | SE |
| $\beta_{0}$ | 3.55 | 0.428 |  | 0.94 | 0.239 |  | 2.06 | 0.215 |  | 2.86 | 1.238 |  | 6.69 | 0.691 |
| $\beta_{z}$ | -0.31 | 0.015 |  | 0.06 | 0.027 |  | 2.32 | 0.504 |  | 0.60 | 0.106 |  | - | - |
| $\beta_{N}$ | -0.12 | 0.031 |  | -0.03 | 0.071 |  | -0.05 | 0.015 |  | -0.13 | 0.079 |  | 0.02 | 0.017 |
| Stage of Development | - | - |  | -- | - |  | - | - |  | - | - |  | -0.01 | 0.007 |
| Mesocosm | 0.16 | 0.395 |  | - | - |  | - | - |  | 0.17 | 0.417 |  | - | - |
| Drainage | - | - |  | - | - |  | - | - |  | 0.04 | 0.193 |  | 0.80 | 0.896 |
| Residual Variance | 0.45 | 0.669 |  | - | - |  | - | - |  | - | - |  | 0.17 | 0.412 |
| For the statistical analyses, the parameter $\varphi$ was set to 0. The quantity $N\int\frac{x^{\varphi}}{z^{\varphi}}p\left( x \right)dx$ was then calculated for each individual in each mesocosm. Parameters for each vital rate were obtained using either linear mixed or generalized linear mixed models. Equations for each vital rate can be found in Table S1. For mean growth, survival and probability of reproduction, standard length was centered on 18mm prior to analysis. Survival and probability of reproduction were fit using binomial errors. Number of offspring was fit with a quasi-Poisson distribution with estimated dispersion parameter of 1.43. | | | | | | | | | | | | | | |

Figure S1. Relationship between asymmetry of length-based competitive interactions ($\boldsymbol{\varphi}$) and ecological quantities (population size and mean body length) and mean and variance in evolutionary rates when $\boldsymbol{\varphi}$ is changed in all the vital rates. $\boldsymbol{\varphi}$ is a measure of the degree of asymmetry in competitive interactions. Negative values of $\boldsymbol{\varphi}$ indicate that smaller sized individuals have a competitive advantage over larger sized individuals. Zero means that competitive ability does not depend on the trait value (symmetrical competition). $\boldsymbol{\varphi}$ values greater than zero indicate that larger individuals are competitively superior to smaller individuals. Measures below the bifurcation for the mean and variance in the evolutionary rates are omitted because their calculation is not well-defined for cyclical dynamics. See text and Table 1 for calculating the trait-based variances


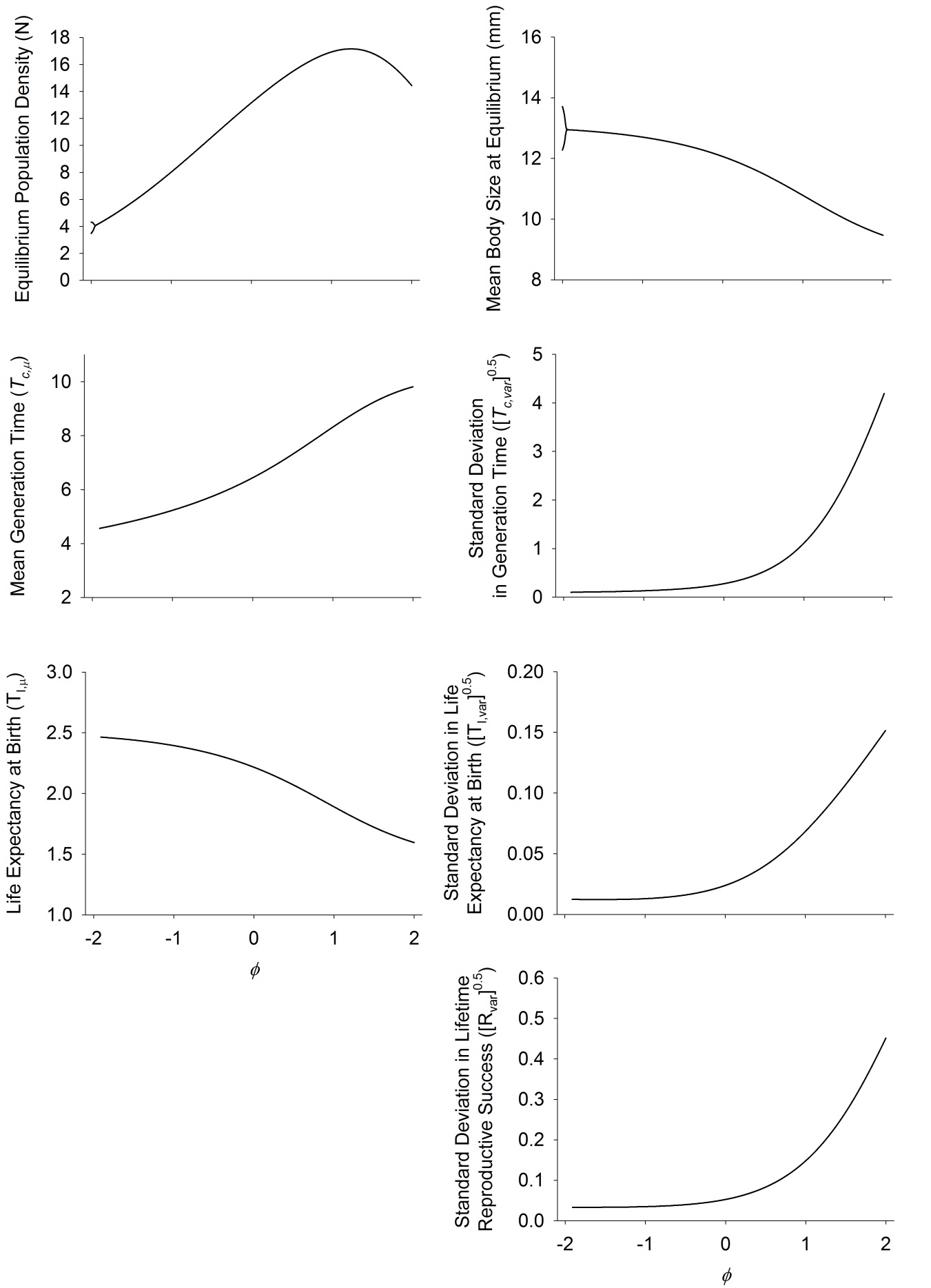


**Supplementary Materials**

**THE EFFECTS OF Asymmetric COMPETITION ON THE LIFE HISTORY OF TRINIDADIAN GUPPIES**

Ronald D. Bassar^1,†^, Dylan Z. Childs^2^, Mark Rees^2^, Shripad Tuljapurkar^3^, David Reznick and Tim Coulson^1^

^1^ Department of Zoology, South Parks Road, University of Oxford, OX1 3PS

^2^ Department of Animal and Plant Sciences, University of Sheffield

^3^ Department of Biology, Stanford University, Palo Alto, California

^4^ Department of Biology, University of California, Riverside, California

^†^ corresponding author – email: ronald.bassar@zoo.ox.ac.uk

**Growth Simulation**

Below is R (R Core Development Team 2012) code to simulate growth of guppies in a factorial design with density crossed with mean body length and estimate growth parameters using the bbmle package.

rm(list=ls(all=TRUE))

#Load necessary libraries

require(bbmle)

require(abind)

#===============================================================

#Simulate Population and Somatic Growth

#===============================================================

#This simulates a 2 x 2 factorial experiment where density is crossed with the mean size-structure

#Note that the size-structure is not normally distributed, but approximates the size distribtion in guppies.

LowDens <- 6

HighDens <- 12

n.pops<-256 #Number of populations

n.guppies.pop <-rep(c(LowDens,HighDens),n.pops/2)#Number of guppies in populations. Either 6 or 12.

n.guppies <- sum(n.guppies.pop) #Total N of guppies

pop <- rep(1:n.pops,n.guppies.pop) #Assign population number

size <- rep(NA,n.guppies) #Vector of sizes

# This makes all the sizes in the population identical and adds whatever value (meanAdjust) to the mean of the size distribution for half

# Use this in conjunction with changing the error on the parameters themselves, which allows for variation among individuals

adult.mean.size <- 15

baby.mean.size <- 8

sd.adult <- 3

sd.baby <- 1

meanAdjust <- 4

error <- 0.01 #This is the error of the parameters for the simulation.

# There are a couple ways to replicate the populations. The first is to assume that replicate populations have exactly the same sizes.

# The second is that there is some variation in the sizes among replicate populations. Choose which to use by changing 'diff.sizes'.

# If F, then the first case, if T, the will use different sizes.

diff.sizes <- T

if (diff.sizes==F){

rand.sizes.babies <- sort(rnorm(4,baby.mean.size,sd.baby))

rand.sizes.adults <- sort(rnorm(2,adult.mean.size,sd.adult))

babiesLowDens <- sort(rand.sizes.babies)

babiesHighDens <- sort(c(rand.sizes.babies,rand.sizes.babies))

juvAdsLowDens <- sort(rand.sizes.adults)

juvAdsHighDens <- sort(c(rand.sizes.adults,rand.sizes.adults))

for(i in 1:n.pops){

if (i<=(n.pops/2)){

if (n.guppies.pop[i]==LowDens){

size[pop==i] <- (abind(babiesLowDens,juvAdsLowDens,along=1))

}

if (n.guppies.pop[i]==HighDens){

size[pop==i] <- (abind(babiesHighDens,juvAdsHighDens,along=1))

}

}

if (i>(n.pops/2)){

if (n.guppies.pop[i]==LowDens){

size[pop==i] <- (abind(babiesLowDens+meanAdjust,juvAdsLowDens+meanAdjust,along=1))

}

if (n.guppies.pop[i]==HighDens){

size[pop==i] <- (abind(babiesHighDens+meanAdjust,juvAdsHighDens+meanAdjust,along=1))

}

}

}

}

if (diff.sizes==T){

for(i in 1:n.pops){

babiesLowDens <- sort(rnorm(4,baby.mean.size,sd.baby))

babiesHighDens <- sort(rnorm(8,baby.mean.size,sd.baby))

juvAdsLowDens <- sort(rnorm(2,adult.mean.size,sd.adult))

juvAdsHighDens <- sort(rnorm(4,adult.mean.size,sd.adult))

if (i<=(n.pops/2)){

if (n.guppies.pop[i]==LowDens){

size[pop==i] <- (abind(babiesLowDens,juvAdsLowDens,along=1))

}

if (n.guppies.pop[i]==HighDens){

size[pop==i] <- (abind(babiesHighDens,juvAdsHighDens,along=1))

}

}

if (i>(n.pops/2)){

if (n.guppies.pop[i]==LowDens){

size[pop==i] <- (abind(babiesLowDens+meanAdjust,juvAdsLowDens+meanAdjust,along=1))

}

if (n.guppies.pop[i]==HighDens){

size[pop==i] <- (abind(babiesHighDens+meanAdjust,juvAdsHighDens+meanAdjust,along=1))

}

}

}

}

#===============================================================

#Function to generate growth data

#===============================================================

predictor <- function(v0,bz,bN,phi){

for(i in 1:n.pops){

p.size <- size[which(pop==i)]

comp.length <- (1/p.size^rnorm(1,phi,abs(phi*error)))%*%t(p.size^rnorm(1,phi,abs(phi*error)))

alphaN <- rowSums( comp.length )

if (i==1){

long.version <- c(alphaN)

}

if (i>1){

long.version <- c(long.version,alphaN)

}

}

u <- rnorm(1,v0,abs(v0*error)) + rnorm(1,bz,abs(bz*error))*(size-sizeoffset) + rnorm(1,bN,abs(bN*error))*long.version

return(u)

}

#===============================================================

#Create likelihood function

#===============================================================

LogLik <- function(v0.hat,bz.hat,bN.hat,phi.hat,sigmahat) {

for(i in 1:n.pops){

p.size <- size[which(pop==i)]

comp.length <- (1/p.size^phi.hat)%*%t(p.size^phi.hat)

alphaN <- rowSums( comp.length )

if (i==1){

long.version <- c(alphaN)

}

if (i>1){

long.version <- c(long.version,alphaN)

}

}

lin.pred <- v0.hat + bz.hat*(size-sizeoffset) + bN.hat*long.version

loglik <- -sum(dnorm(growth,lin.pred,sigmahat,log=TRUE))

return(loglik)

}

#===============================================================

#Run simulation and estimate parameters

#===============================================================

sizeoffset <- 18 #Centered size, i.e. the size at which the intercept is estimated.

v0 <- 3.55

bz <- -0.31

bN <- -0.09

phi <- 1.75

growth <- predictor(v0,bz,bN,phi) #Creates data based on known parameters and error

plot(size,growth) #plots growth

start = list(v0.hat=8,bz.hat=-0.6,bN.hat=-0.32,phi.hat=0.5,sigmahat=1) #Starting values for estimation

test <- mle2(minuslogl = LogLik,start=start)

summary(test)

#===============================================================

#Plot Fits

#===============================================================

plot(size,growth, typ='p', col="black", cex.lab = 1.5, cex = 1.5, xlab="Trait Value z", ylab="Growth")

for(i in 1:n.pops){

p.size <- size[which(pop==i)]

comp.length <- ((1/p.size)%*%t(p.size) )^coef(test)['phi.hat']

alphaN <- rowSums( comp.length )

long.version <- c(alphaN)

l <- coef(test)[1] + coef(test)[2]*(p.size-sizeoffset) + coef(test)[3]*long.version

lines(p.size,l, lty='dashed', col="blue")

}
